# Evaluating adherence, tolerability and safety of oral calcium citrate in elderly osteopenic subjects: a real-life non-interventional, prospective, multicenter study

**DOI:** 10.1007/s40520-024-02696-9

**Published:** 2024-02-12

**Authors:** Mariangela Rondanelli, Salvatore Minisola, Marco Barale, Daniele Barbaro, Francesca Mansueto, Santina Battaglia, Gloria Bonaccorsi, Santina Caliri, Alessandro Cavioni, Luciano Colangelo, Sabrina Corbetta, Federica Coretti, Giorgia Dito, Valentina Gavioli, Ezio Ghigo, Raffaele Giannattasio, Paola Lapi, Blas Maiorana, Costanza Marra, Maurizio Mazzantini, Elisabetta Morini, Fabrizio Nannipieri, Vincenzo Nuzzo, Fabiana Parri, Simone Perna, Rachele Santori, Massimo Procopio

**Affiliations:** 1 Department of Public Health, Experimental and Forensic Medicine, University of Pavia, Via Forlanini, 2, 27100 Pavia, Italy; 2 Department of Clinical, Internal, Anesthesiological and Cardiological Sciences, “Sapienza” University of Rome, Rome, Italy; 3 Division of Endocrinology, Diabetology and Metabolic Diseases, Department of Medical Sciences, Molinette Hospital, University of Turin, Turin, Italy; 4 Sezione Dipartimentale Aziendale di Endocrinologia Ospedale di Livorno, Livorno, Italy; 5 Clinical Research, Abiogen Pharma, Pisa, Italy; 6 Department of Translational Medicine, Menopause and Osteoporosis Center, University of Ferrara, Ferrara, Italy; 7 IRCCS, Servizio di Endocrinologia, Centro Neurolesi Bonino Pulejo, Messina, Italy; 8 Bone Metabolism Diseases and Diabetes Unit, IRCCS Istituto Auxologico Italiano, Milan, Italy; 9 Department of Biomedical, Surgical and Dental Sciences, University of Milan, Milan, Italy; 10 UOSD Malattie Endocrine, del Ricambio e della Nutrizione Ospedale del Mare ASL Napoli1 Centro, Naples, Italy; 11 Endocrinoly and Diabetology Service, IRCCS Ospedale Galeazzi Sant’Ambrogio, Milan, Italy; 12 Ospedale San Gennaro, ASL Napoli 1 Centro, Naples, Italy; 13 Policlinico di Foggia, Reparto di Ortopedia E Traumatologia Universitaria, Foggia, Italy; 14 Rheumatology Unit, Azienda Universitario Ospedaliera di Pisa, Pisa, Italy; 15 Division of Human Nutrition, Department of Food, Environmental and Nutritional Sciences (DeFENS), Università Degli Studi di Milano, Milan, Italy

**Keywords:** Osteoporosis, Calcium citrate, Supplementation, Adherence, Adverse reaction

## Abstract

**Background:**

Osteoporosis is a common concern in the elderly that leads to fragile bones. Calcium supplementation plays a crucial role in improving bone health, reducing fracture risk, and supporting overall skeletal strength in this vulnerable population. However, there is conflicting evidence on the safety of calcium supplements in elderly individuals.

**Aim:**

The aim of this study was to evaluate the adherence, safety and tolerability of calcium citrate supplementation in elderly osteopenic subjects.

**Methods:**

In this non-interventional, prospective, multicenter study, subjects received daily 500 mg calcium citrate supplementation for up to one year. Adherence was calculated based on compliance and persistence. Safety was assessed through adverse reactions (ARs), deaths, and clinical laboratory evaluations.

**Results:**

A total of 268 Caucasian subjects (91.4% female, mean age 70 ± 4.5 years) participated in the study. Mean adherence to treatment was 76.6 ± 29.5% and half of subjects had an adherence of 91% and ~ 33% of participants achieved complete (100%) adherence. ARs were reported by nine (3.9%) subjects, primarily gastrointestinal disorders, with no serious ARs. The frequency of all adverse events (including ARs) was significantly higher in subjects with adherence of < 80% (41.6%; 32/77) vs. those with adherence ≥ 80% (11%; 16/145, p < 0.0001). Both systolic and diastolic blood pressure decreased from baseline to follow-up visit (change of -2.8 ± 13.9 mmHg, p = 0.0102 and -2.1 ± 10.4 mmHg, p = 0.0116, respectively).

**Conclusion:**

This study demonstrated favorable adherence to calcium citrate supplementation in elderly osteopenic subjects. The occurrence of ARs, though generally mild, were associated with lower adherence to calcium supplementation.

**Supplementary Information:**

The online version contains supplementary material available at 10.1007/s40520-024-02696-9.

## Introduction

The occurrence of fractures and osteoporosis are significant concerns in elderly adults, as ageing remains one of the primary risk factors for these conditions [1]. While the incidence of fracture and risk may vary across different populations, geography, ethnicities, and socioeconomic status, the incidence of fragility fractures significantly increases with advancing age, particularly after the age of 50 years [2]. However, the prevalence of osteoporosis rises as individuals age, with approximately 10% of women at 60 years, 20% at 70 years, 40% at 80 years, and two-thirds of women at 90 years affected by this condition. Alarmingly, about half of all fractures occur after the age of 75 [2]. This highlights the critical importance of addressing osteoporosis and fracture prevention in older adults. Calcium supplementation, usually with vitamin D, is also a required complement to other specific pharmacological treatments of osteoporosis because these supplements have been an inherent part of the therapies of osteoporosis as evaluated in clinical trials [3, 4]. Different forms of calcium salts are available, but the products containing calcium citrate and calcium carbonate complexes are the most frequently used. A meta-analysis [5], based on 15 studies involving 184 subjects, showed that calcium citrate was absorbed to a greater extent than calcium carbonate (approximately 22–27% more), both on an empty stomach and with a full stomach. Based on these findings, calcium citrate should be considered the preferred choice, even in patients with achlorhydria, a common condition in the elderly, as well as in individuals with inflammatory bowel disease or absorption disorders. Calcium citrate supplements should also be recommended for individuals treated with H2-antagonists or proton pump inhibitors [6]. Furthermore, individuals residing in assisted healthcare facilities and those engaged in work activities that make it difficult to consume calcium with meals should use calcium citrate, as it can be taken with or without food [7]. Moreover, individuals with medical conditions and older individuals can also derive significant benefits from the use of calcium citrate. As it is well known, however, adherence to therapy plays a crucial role in the success of any treatment. It refers to the extent to which patients follow their prescribed medication regimen or treatment plan. High adherence improves treatment effectiveness, enhances patient outcomes, and reduces the risk of complications or treatment failure. On the other hand, poor adherence can lead to suboptimal results and decreased therapeutic benefits. Therefore, maintaining high levels of adherence is essential for maximizing the potential benefits of any therapy and ensuring its success in achieving the desired health outcomes [8].

Historical data on the safety of calcium supplementation has yielded conflicting results. Two meta-analysis studies by Bolland et al. suggested an increased risk of cardiovascular events, specifically myocardial infarction (MI), among calcium users. However, other studies or critical reviews of the literature by other groups of researchers do not support this finding [9–11]. As a consequence, the safety of calcium supplements remains a topic of controversy, particularly as their usage becomes more prevalent. Therefore, it is essential to investigate the safety profile of calcium citrate in these populations.

This non-interventional, prospective, multicenter study evaluated the adherence, tolerability, and safety of calcium citrate administration in an "outpatient" population within the context of routine clinical practice.

## Methods

### Study design

This was a non-interventional, prospective multi-centre, study. A total of 277 elderly osteopenic subjects (aged 65–80 years) were screened across 11 centres throughout Italy: Molinette, Torino, Azienda di Servizi alla Persona, Università di Pavia; Menopause and Osteoporosis Center, University of Ferrara; Umberto I, Roma; AOU, Pisa; Osp. Del Mare, Napoli; San Gennaro, Napoli; Ospedale Galeazzi Sant’Ambrogio, Milano; Osp. Riuniti, Foggia; Osp. Riuniti, Livorno and Bonino Pulejo, Messina. This study was performed from April 2019 to June 2022. Subjects were screened for the study during specialist’s visit and at the moment they first received advice to start calcium citrate supplementation from their physician as per their clinical practice. The inclusion criteria for the study were as follows: individuals with osteopenia according to definition from the World Health Organisation[12, 13] (T-score <  −  1.0 and > − 2.5 at any sites measured by dual-energy X-ray absorptiometry; DXA, Hologic or Lunar) [12, 13], aged between 65 and 80 years, individuals requiring calcium supplementation according to physician’s judgement, individuals capable of understanding the study procedures and adhering to the protocol requirements, and individuals legally capable of providing written informed consent for participation in the study (signed and dated by the interested party). Exclusion criteria were individuals who had received calcium supplementation within the previous 12 months or had a T score < − 2.5. This study was approved by the Ethics Committee of the Fondazione IRCCS Policlinico San Matteo, Pavia, Italy (approval number N. 20180107341) and performed in accordance with the ethical standards as laid down in the 1964 Declaration of Helsinki.

### Treatment

The daily dietary supplement was formulated as sticks, with each stick containing 500 mg of calcium citrate dissolved in 10 ml of solution (Calciobase, Abiogen Pharma S.p.A., Pisa, Italy). During the study, participants were allowed to continue taking their regular medications alongside Calciobase. The ingredients of Calciobase included water, calcium citrate, xanthan gum (a stabilizer), lactic acid (an acidity regulator), potassium sorbate (a preservative), flavoring agents, and sucralose (a sweetener). Calciobase does not contain any added sugars, gluten, or lactose, making it suitable for individuals with dietary restrictions.

### Data collection

After enrolment at the first visit subjects could return at the physician's discretion. During these visits, the data available for each visit were collected within 12 months according to standard clinical practice.

During the initial visit, the study was explained by the physician and the informed written consent to the study was collected. The following procedures were performed: collection of demographics (gender, date of birth, height (cm), weight (kg), race; vital signs (systolic blood pressure/diastolic blood pressure, SBP/DBP (mmHg), heart rate, HR (bpm), respiration rate (bpm) and body temperature (°C); physical examination (general conditions, nutritional status, circulatory system, respiratory system, abdomen, lymph nodes, skin and appendages, mucous membranes, muscles/skeleton, neurological system, other); medical history; concomitant therapies; laboratory tests eventually carried out in the previous thirty days or in the thirty days following the visit were recorded, in particular those recommended as per SIOMMMS guidelines for osteoporosis [14]; delivery and explanation of the Subject’s Diary.

During the second visit or further visits, V2 (within 12 months after V1), the following procedures were performed: registration and evaluation of adverse reactions (AR) reported by the subject and / or on the visit; vital signs (as in V1); physical examination (as in V1); concomitant therapies; laboratory tests carried out in the previous two weeks or in the two weeks following the visit can be evaluated (same parameters as V1); urine analysis (the same as in V1); recording of results of instrumental examinations such as BMD, X-ray imaging, if performed and checking of the contents of the subject’s diary. In any additional visits, carried out during clinical practice between the initial visit and the following 12 months, the procedures described for Visit 2 were performed.

The events described by the subject in the subject’s diary were evaluated and associated to calcium supplementation. If the correlation was confirmed, the event was registered into medical records as ARs. If the correlation was not confirmed, the event was registered into medical records as adverse events (AEs).

Data concerning safety and tolerability, other than data concerning healthy status of the subject (as vital signs, physical examination, concomitant therapies and laboratory test results, if available) were described in the medical records.

## Study endpoints

### Primary outcome measure

The primary objective of the study was to assess the adherence to calcium citrate supplementation in elderly subjects aged 65–80 years. Adherence was calculated as the arithmetic mean of both compliance and persistence as described in detail elsewhere [15]. Briefly, compliance was calculated as the percentage ratio between the number of sticks taken with and the number of sticks that must be taken according to the physician's prescription. The percentage persistence was calculated as the number of days the integrator was taken over the prescribed duration. Data for primary endpoints were obtained from the subjects' diaries. The prescribed dose was determined by multiplying the prescribed number of sticks per day by the prescribed duration. If the final visit occurred before the prescribed duration, the duration was calculated as the difference between the date of the final visit and the start date of the supplementation. In some cases, the end date was determined based on the date of a phone call.

### Secondary outcomes

The secondary objective of the study was to evaluate the safety and tolerability of calcium citrate in the same elderly osteopenic subjects. The correlation between calcium supplementation and all AEs reported in the subjects' diaries was documented in the medical records, and all ARs were entered into the electronic Case Report Form (e-CRF). Any serious ARs, including those leading to withdrawal, were described in detail. Laboratory and urinalysis parameters were recorded at each visit, and changes from baseline in these parameters were documented for patients in the safety population. The laboratory and urinalysis parameters were used to determine the number and percentage of individuals with measurements classified as normal or abnormal. ARs (a subgroup of AEs) and laboratory tests were assessed following the normal clinical practices of each site. Data for secondary endpoints were collected from the subjects' medical records and diaries. ARs resulting from the supplement application and any other special situations that occurred during the study were described. ARs and special situations were coded using the Medical Dictionary for Regulatory Activities (MedDRA®). Individual listings and summary tables were used to present the following information, categorized by expectedness:

- The number of ARs, including recurring reactions within non-overlapping time periods. For subjects experiencing the same reaction multiple times, the frequency of occurrence was recorded. The mean cumulative duration was calculated for each AR.
- The number and percentage of subjects with at least one recurrence of a specific AR, disregarding any repeated occurrences.
- The number and percentage of subjects experiencing at least one AR.

Analysis of laboratory and urinalysis parameters, vital signs, and physical examination parameters were also included as secondary endpoints.

### Statistical analysis

All parameters measured in this study were analyzed using classic descriptive statistics, including mean, standard deviation (SD), minimum and maximum values for quantitative variables, and frequencies for qualitative variables.

Data generated in this study were recorded in a study-specific electronic system developed on the SAS® PheedIt platform. After data entry, resolution of discrepancies, and completion of data collection, the database was locked to prevent further modifications. Following quality checks, the SAS format database was used for statistical analysis.

For the statistical analysis of adherence, only subjects who received calcium citrate supplementation and completed the baseline visit and at least one post-baseline assessment, returning the diary, the so called "Intention to Treat Population" (ITT population), were included. All subjects who received at least one calcium citrate supplementation, regardless of post-baseline assessments, instead, were considered part of the "Safety Population" and included in safety and tolerability analyses.

Comparison between normally distributed (continuous) variables was performed by Mann Whitney U test or t-test and differences in frequencies (categorical variables) was performed by Fisher exact test. Differences between paired non-parametric (nominal data) were assessed using the McNemar test. Associations between 2 variables at a time with adherence was performed by Pearson correlation coefficient and the association between a range of potential predictor variables (age, gender, BMI, SBP, concomitant medication, vitamin D supplementation and the presence of all AEs) with adherence was assessed by multivariate logistic regression analysis.

The sample size calculation was based on the primary endpoint of the study, which was the proportion of subjects with adherence ≥ 80%. Assuming a two-tailed probability of Type I error of 0.05, a sample size of 217 subjects would provide 98% power to detect a 13% difference between the null hypothesis (57% adherence) and the alternative hypothesis (70% adherence). The null hypothesis was based on a previous study [16]. Accounting for approximately 30% non-evaluable subjects, a total of 300 subjects were included in the observational study. A p value of < 0.05 was considered statistically significant. Statistical analysis was conducted using SAS® version 9.4 or higher for Windows® (SAS Institute Inc., Cary, North Carolina, USA).

## Results

### Baseline clinical characteristics

**Fig. 1:**
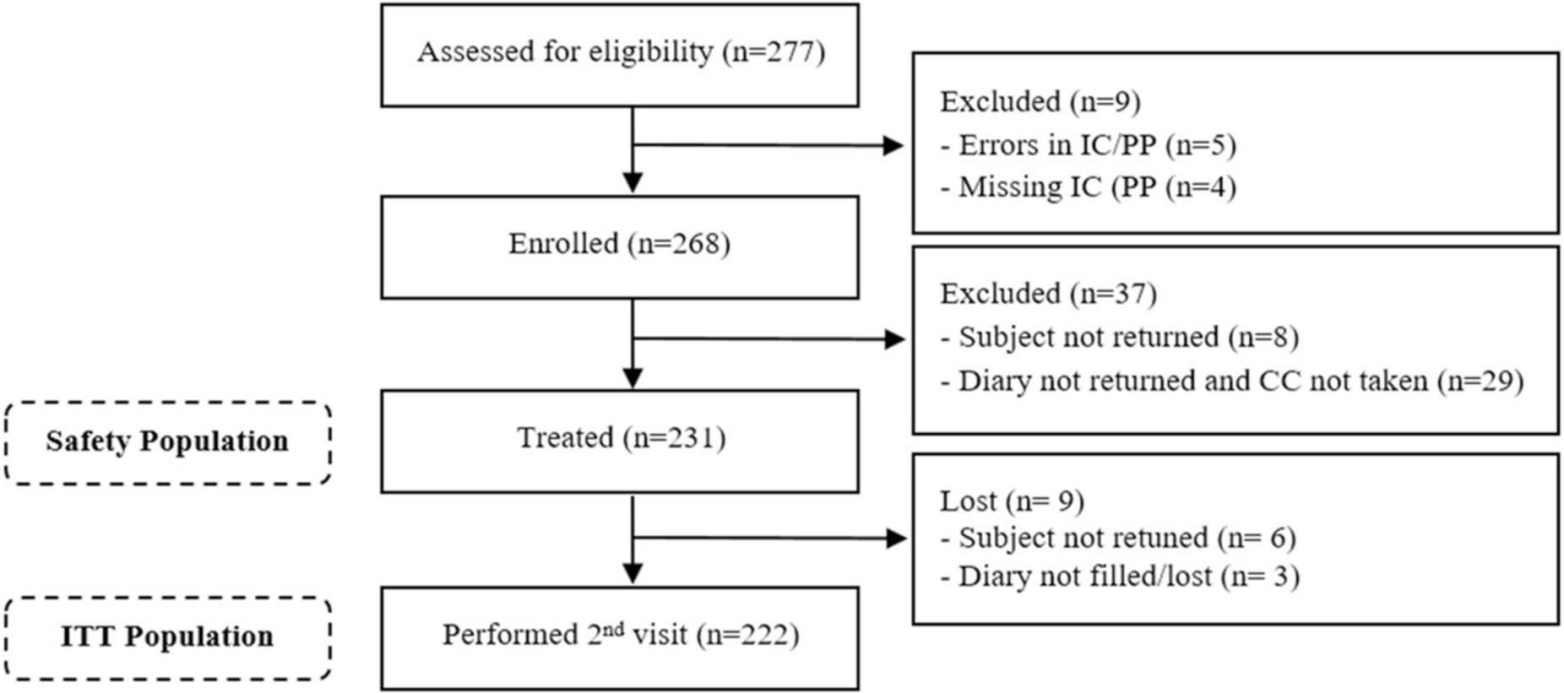
Subject disposition. *CC* calcium citrate, *IC* informed consent, *PP* privacy policy

Out of 277 subjects who underwent screening, a total of 268 (96.8%) individuals were successfully enrolled in the trial. Among screened subjects, 9 were excluded from the study due to incomplete or incorrect filling of their informed consent and/or privacy information (Fig. 1). Among the remaining subjects, 37 individuals either declared that they had never taken calcium citrate or failed to return for the control visit or respond to telephone contact. As a result, these 37 subjects were deemed ineligible for evaluation in terms of both efficacy and safety. Additionally, nine subjects did not provide diary records but stated that they had taken calcium citrate. Therefore, these nine individuals could only be evaluated for safety (Safety Population, N = 231) and a total of 222 subjects comprised the intention-to-treat (ITT) population (Fig. 1).

The study population (N = 268) comprised 245 females (91.4%) and 23 males (8.6%). Participants had a mean age of 70.0 ± 4.5 years and mean BMI of 26.5 ± 4.9 kg/m^2^ (Table 1). Among the enrolled subjects, 4 subjects were less than 65 years of age, as specified in the inclusion criteria, with ages of 38, 57, 63, and 63 years. In the ITT population, 51.4% (N = 114) of subjects reported using more than three concomitant therapies, while 48.6% (N = 108) reported using less than three concomitant therapies, and 14.9% (N = 33) reported not taking any other medication. Additionally, 59.5% (N = 132) of individuals reported taking vitamin D3 supplementation. At baseline, medical anomalies were detected in 28.6% (N = 42) of the subjects as described in Table 1.

**Table 1:** Baseline characteristics of subjects

Characteristic	N	
General		
Age, years	268	70.0 ± 4.5
Female, n (%)	268	245 (91.4)
Caucasian, n (%)	268	268 (100)
BMI (kg/m^2^)	251	26.5 ± 4.9
Vital signs		
Systolic blood pressure (mmHg)	166	130.7 ± 16.9
Diastolic blood pressure (mmHg)	166	79.5 ± 8.7
Heart rate (bpm)	149	76.2 ± 9.1
Concomitant medications		
≥ 3 medications, n (%)	222	114 (51.4)
Had < 3 medications, n (%)	222	108 (48.6)
Had no other medication, n (%)	222	33 (14.9)
Vitamin D supplementation, n (%)	222	132 (59.5)
Comorbid diseases		
Total medical anomalies, n (%)	147	42 (28.6)
Muscle/skeleton disorders, n (%)	147	17 (11.6)
Circulatory systems disorders, n (%)	147	8 (5.4)
Gastroesophageal reflux, n (%)	147	3 (2.0)
Other disorders	147	13 (4.1)

Data are presented as number and % or mean and SD. N represents the number of subjects where data was available. Percentages (%) are based on the number of subjects with data

*BMI* body mass index

### Adherence to treatment

**Fig. 2:**
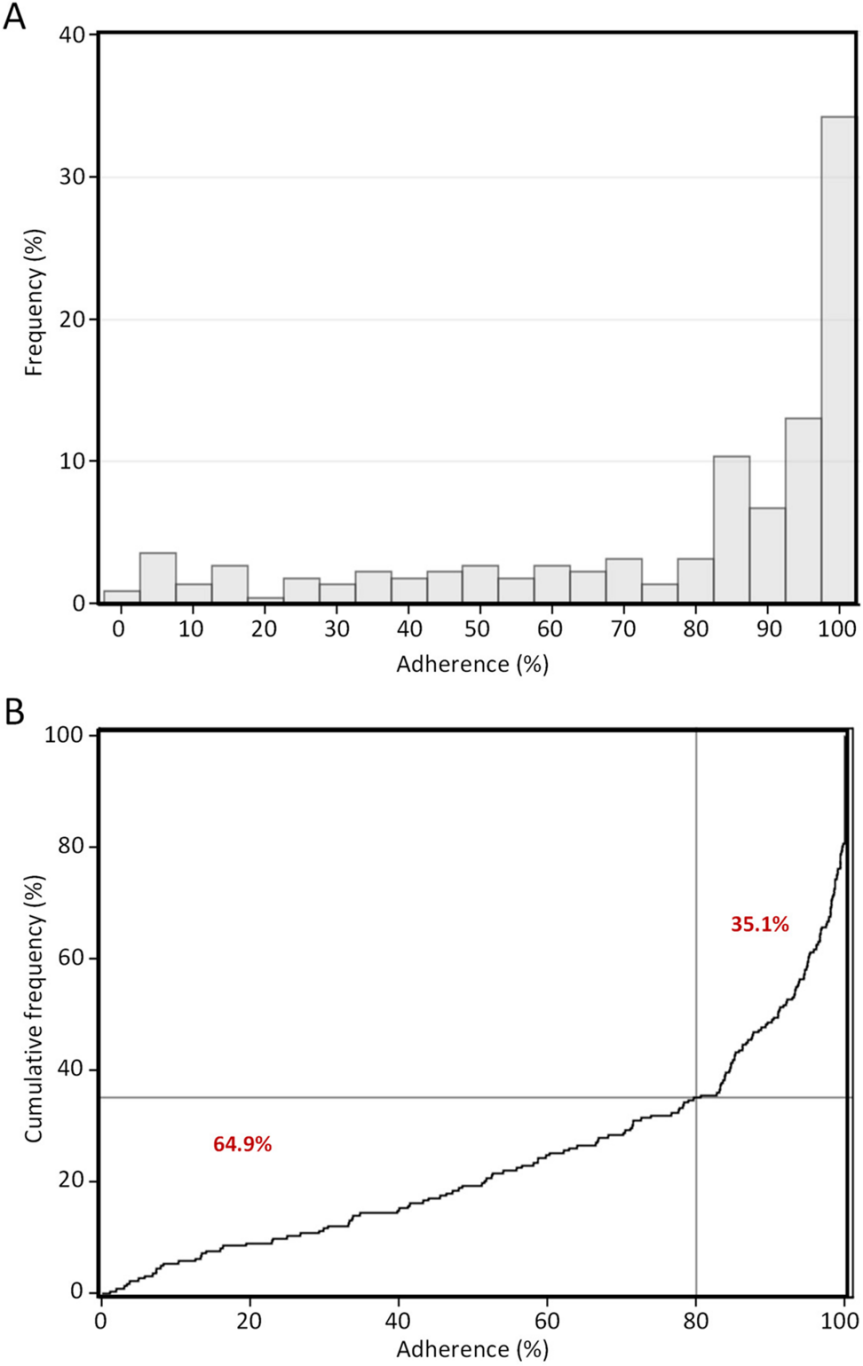
Frequency, frequency %, and cumulative frequency distribution of adherence in ITT population

In this study, we observed an average adherence to oral calcium citrate supplementation of 76.6 ± 29.5%, with a median of 91.0%. Specifically, half of subjects had an adherence of at least 90% while approximately one-third of individuals had an adherence of 100% (Fig. 2A). In the cumulative distribution graph (Fig. 2B), it can be observed that 64.9% of subjects achieved an adherence rate of ≥ 80% (35.1% not achieving adherence of ≥ 80%), which was higher than the reference rate of 57% (p = 0.0179), previously reported [16]. In univariate analysis, adherence was also found to be weakly correlated with BMI (*r* = 0.18; p = 0.0097; Supplementary Table S1), but not with other variables such as age, the total number of medications received and SBP. Stratifying subjects by adherence (< 80% vs. ≥ 80%,), we observed a higher frequency of AEs reported in subjects with adherence < 80% vs. those having an adherence ≥ 80% (32/77; 41.6% vs. 16/145, 11%. p < 0.0001). In multivariate logistic regression analysis, the absence of AEs was associated with a 6.1-fold increase in achieving ≥ 80% adherence (OR 6.07, 95% CI 2.8–13.1, p < 0.0001) but not with other variables examined (Table 2).

**Table 2:** Multivariate linear logistic regression analysis of variables associated with achieving ≥ 80% adherence

Characteristic	*β-*coefficient	SE	OR (95% CI)	*p* value
Age (< 70 vs. ≥ 70 years)	0.11	0.35	1.12	0.74
BMI (< 30 vs. ≥ 30 kg/m^2^)	0.22	0.44	1.25	0.61
Gender (male vs. female)	1.26	0.81	3.5	0.12
Medication (< 3 vs. ≥ 3 treatments)	0.25	0.41	1.29	0.53
No adverse event (yes/no)	1.8	0.39	6.07	** < 0.0001**
SBP (< 130 vs. ≥ 130 mmHg)	0.08	0.35	1.09	0.81
Vitamin D supplementation (yes/no)	0.29	0.41	1.34	0.48

Statistically significant p-values are represented by bold text

*BMI* body mass index, *CI* confidence interval, *OR* odds ratio, *SBP* systolic blood pressure, *SE* standard error

### Safety and tolerability

The secondary objective of the study was to assess the safety and tolerability of calcium citrate.

Overall, a total of 20 AR episodes (mean duration of 14.2 ± 19.3 days) were reported of which 19 (95%) were of gastrointestinal origin. Constipation was the most frequently reported gastrointestinal disorder, accounting for 50% of all ARs. Additionally, one AR episode was related to nutrition disorders, specifically decreased appetite, with an average and median duration of 6 days (Table 3).

**Table 3:** Episodes of adverse reactions and their duration in days

Adverse reactions	N (%)	Mean ± SD (days)
Gastrointestinal disorders		
Abdominal discomfort	1 (5)	6.0
Abdominal pain (upper)	2 (10)	3.5 ± 3.5
Constipation	10 (50)	8.1 ± 16.6
Dyspepsia	2 (10)	47.5 ± 10.6
Dysphagia	1 (5)	4.0
Flatulence	1 (5)	40.0
Nausea	2 (10)	22.0 ± 25.5
All	19 (95)	14.6 ± 19.7
Nutritional disorders		
Decreased appetite	1 (5)	6.0
All	1 (5)	6.0
All	20 (100)	14.2 ± 19.3

The data are presented as numbers, percentages (%), means and standard deviations (SD), and medians with minimum and maximum values. *N* number of AR episodes, *SD* standard deviation

There were no serious ARs during the treatment period. A total of 9 subjects (3.9%) experienced at least one AR. Among these cases, 5 individuals experienced one episode each, 1 person had two episodes, and 3 individuals encountered three episodes (Supplementary Table 2).

In a small subset of individuals (N = 16) where blood samples were taken both at baseline and follow up visit, significant variations in specific hematological parameters were observed. While levels of neutrophils (3.48 ± 2.14%, p = 0.0105), and MCH (0.88 ± 0.51 pg/cell, p = 0.0420) increased, lymphocyte (3.75 ± 3.04%, p = 0.0295), and platelet count (27.2 ± 20.3 × 10^3^/µL, p = 0.0220) were observed to decrease.

In addition to evaluating the frequency of ARs, all AEs were evaluated. There were 50 (21.6%) subjects with at least one non-related AE (Supplementary Table 2). Out of a total of 110 episodes, 79.1% of reported suspected non-related AEs categorized as gastrointestinal disorders (Supplementary Table 3).

### Vital signs, physical findings, and other observations related to safety

Differences were also observed among a range of vital signs comparing baseline a post-baseline values (Supplementary Table 4). In a subset of individuals where SBP was measured (N = 65), mean SBP was observed to decrease from 130.7 ± 16.9 mmHg to 127.9 ± 14.5 mmHg (p = 0.0102) from baseline to V2. Likewise, DBP was also seen to decrease from 79.5 ± 8.7 mmHg to 77.4 ± 8.6 mmHg (baseline to V2) (p = 0.0116). No significant variation in heart rate, respiratory rate and body temperature were observed. Significant positive variations were also observed in the frequency of normal/abnormal signs or symptoms for other variables such as nutritional status (p = 0.0116), circulatory system disorders (p = 0.0001) and muscles/skeleton disorders (p = 0.0067) from baseline to post-baseline visit (Supplementary Table 5).

## Discussion

This non-interventional, prospective, multicenter study provides novel insights into the adherence and safety of calcium citrate supplementation in an elderly osteopenic population. The primary objective of the study, to evaluate adherence in this population was achieved; mean adherence rate was 76.6 ± 29.5%, with a median of 91.0% observed up to 1 year, indicating satisfactory adherence overall. Moreover, approximately one-third of the subjects achieved 100% adherence, reflecting a high level of commitment to this supplementation regimen. Importantly, AR episodes to calcium supplementation were reported by a low percentage of participants (3.9%) and were strictly related to gastrointestinal disorders, with no serious ARs observed. As expected, in subjects achieving an adherence of ≥ 80%, a lower rate of AEs was reported compared to subjects with an adherence < 80% where the rate of AEs was 41.6%. This association was also confirmed through multivariate logistic regression analysis, which also demonstrated no significant association between calcium citrate supplementation and concomitant vitamin D assumption.

As for any treatment, adherence is the most important factor affecting supplementation effectiveness. Results from community based trials in which the adherence to calcium supplementation was moderate or low have often been negative, whereas studies carried out on institutionalized patients with supervised pill administration resulted in significant benefit [17]. However compliance to long-term calcium and vitamin D supplementation was found to be between 20 and 60% [18, 19]. In our study, we found high levels of adherence among elderly subjects (mean rate: 76.6 ± 29.5%), which is consistent with the findings of the POSSIBLE-US study by Barrett-Connor et al. [20]. In the POSSIBLE-US study, the probability of persisting on calcium therapy (i.e., adherence to treatment) during the first 12 months was over 75% for both subjects using calcium only and those using supplements. It is important to note that adherence to osteoporosis treatment, including calcium supplementation can vary widely. A review by Yeam et al. [8] reported a prevalence of osteoporotic medication adherence ranging from 12.9 to 95.4%. This wide range indicates the diverse nature of adherence rates in different populations and settings. While adherence to calcium citrate supplementation in elderly individuals can be challenging, our study demonstrated high levels of adherence among this population.

There has been some controversy in the literature over the past decade regarding the cardiovascular safety of calcium supplements. Two meta-analyses by Bolland et al. [21, 22] suggested an increased risk of cardiovascular events, particularly MI, in patients taking calcium supplements. However, Lewis et al. [9]) subsequently demonstrated that calcium supplementation primarily increases gastrointestinal (GI) AEs while not significantly impacting upon MI rates. Other studies do not support the notion of cardiovascular risk associated with calcium supplementation [10, 11, 23]. GI disorders related to calcium supplementation, instead, are well described [23, 24]. Our study aligns with these findings, as we did not observe any serious AR, but only 50 AE episodes, predominantly (79.1%) GI related.

Data derived from recent studies consistently indicate that calcium supplements do not pose an elevated cardiovascular risk. The 5-year post-intervention outcomes of The Women’s Health Initiative (WHI) study, as reported by Cauley et al.[25], demonstrated no significant differences in the risk of MI, stroke, or mortality between the calcium/vitamin D group and the placebo group [25]. This finding is supported by additional safety data from various large prospective studies [26, 27]. Given the importance of calcium and vitamin D for maintaining bone health, these findings provide reassurance regarding their safety and efficacy in maintaining bone homeostasis [23].

Adherence was also found to be negatively associated with the presence of AEs. In contrast to this observation, Conti et al. [28] reported a negative impact on adherence during subsequent control visits when AE episodes were associated with calcium and vitamin D supplementation. Nevertheless, the specific type of calcium supplement used in their study was not specified, necessitating further investigation. In this study we found no correlation between AEs and concomitant vitamin D supplementation, suggesting that the use of vitamin D does not contribute to increased AE risk.

An interesting finding that emerged from our analysis was that blood pressure was observed to significantly decrease during the course of the study and the presence of abnormal signs/symptoms from baseline to follow up visit for some specific health indicators, such as nutritional status, circulatory system, and musculoskeletal system were significantly reduced. Our findings align with existing literature, which provides substantial evidence supporting the beneficial effects of calcium supplementation on lipid profiles and blood pressure. A randomized controlled trial (RCT) that included 223 postmenopausal women examined the impact of calcium supplementation versus placebo on lipid profiles. The study revealed that calcium citrate supplementation led to a 7% increase in high-density lipoprotein and a 6% decrease in low-density lipoprotein after one year of follow-up [29]. Other data suggest that calcium supplements may induce transient hypercalcemia and reduce parathyroid hormone (PTH) levels, which could potentially contribute to lowered blood pressure [30, 31]. A systematic review of 42 trials investigating the effect of calcium supplementation on blood pressure demonstrated a reduction in systolic blood pressure by 1.44 mm Hg (95% CI – 2.20 to – 0.68; p < 0.001) and a decrease in diastolic blood pressure of 0.84 mm Hg (95% CI – 1.44 to – 0.24; p < 0.001). These findings are consistent with our own observations, providing additional support for the positive impact of calcium supplementation on lipid profiles and blood pressure, reinforcing the potential benefits of incorporating calcium supplements into one's health regimen. While the BP lowering effects of calcium supplementation are more increasingly recognised, the precise mechanisms by which calcium citrate elicits these effects is not yet fully understood.

## Conclusion

In this study, we observed a high rate of adherence to calcium citrate supplementation over a one-year period in osteopenic elderly subjects. Additionally, the incidence of ARs was low (3.9%), further emphasizing the tolerability of calcium citrate. The reported AEs were relatively few, with a total of 50 cases (21.6%). Importantly, the majority of AEs were of mild intensity (not severe) and GI related. It is important to note that the presence of vitamin D supplementation did not impact upon adherence, suggesting that subjects were able to effectively adhere to both treatments. Future studies designed to assess the long-term impact of calcium citrate supplementation on hard endpoints, such as bone density, fractures/falls, quality of life measures and adherence are needed. These long-term outcomes will provide a more comprehensive understanding of the effects of calcium citrate and its potential benefits in the management of health.

### Supplementary Information

Below is the link to the electronic supplementary material.

Supplementary file1 (DOCX 16 KB)

Supplementary file2 (DOCX 14 KB)

Supplementary file3 (DOCX 19 KB)

Supplementary file4 (DOCX 16 KB)

Supplementary file5 (DOCX 20 KB)

## Data Availability

Data can be made available from the corresponding author upon request.
