# Supplementary material for: Evaluating adherence, tolerability and safety of oral calcium citrate in elderly osteopenic subjects: a real-life non-interventional, prospective, multicenter study
Source: Aging Clin Exp Res. 2024 Feb 12;36(1):38. doi: 10.1007/s40520-024-02696-9 (PMC10861607; doi:10.1007/s40520-024-02696-9)
Supplement: Supplementary file 1 — Supplementary file1 (DOCX 16 KB) [file 40520_2024_2696_MOESM1_ESM.docx]

**Supplementary Table 1.** Pearson correlation matrix between adherence and clinical variables

|  |  | Adherence | Age | BMI | Medication | SBP |
| --- | --- | --- | --- | --- | --- | --- |
| Adherence | *r* |  | 0.058 | 0.18 | 0.086 | -0.023 |
|  | p-value | − | 0.39 | **0.0097** | 0.20 | 0.75 |
|  | *n* |  | *222* | *209* | *222* | *193* |
| Age | *r* | 0.058 |  | -0.11 | 0.23 | 0.15 |
|  | p-value | 0.39 | − | 0.10 | **0.0007** | **0.034** |
|  | *n* | *222* |  | *209* | *222* | *193* |
| BMI | *r* | 0.18 | -0.11 |  | -0.012 | 0.19 |
|  | p-value | **0.0097** | 0.1042 | − | 0.86 | **0.0063** |
|  | *n* | *209* | *209* |  | *209* | *189* |
| Medication | *r* | 0.086 | 0.23 | -0.012 |  | -0.063 |
|  | p-value | 0.20 | **0.0007** | 0.86 | − | 0.38 |
|  | *n* | *222* | *222* | *209* |  | *193* |
| SBP | *r* | -0.023 | 0.15 | 0.20 | -0.063 |  |
|  | p-value | 0.75 | **0.034** | **0.0063** | 0.38 | − |
|  | *n* | *193* | *193* | *189* | *193* |  |

BMI = body mass index, SBP = systolic blood pressure. Statistically significant p-value are in bold text and correlation was assessed using the Pearson Correlation Coefficient (*r*).
