# Supplementary material for: Evaluating adherence, tolerability and safety of oral calcium citrate in elderly osteopenic subjects: a real-life non-interventional, prospective, multicenter study
Source: Aging Clin Exp Res. 2024 Feb 12;36(1):38. doi: 10.1007/s40520-024-02696-9 (PMC10861607; doi:10.1007/s40520-024-02696-9)
Supplement: Supplementary file 2 — Supplementary file2 (DOCX 14 KB) [file 40520_2024_2696_MOESM2_ESM.docx]

**Supplementary Table 2.** Number of adverse events per subject in the safety population (N=231), not related and related to calcium citrate supplementation.

| Frequency | Not related | Related |
| --- | --- | --- |
| 1 | 34 | 5 |
| 2 | 12 | 1 |
| 3 | 2 | 3 |
| 4 | 2 | 0 |
| Total | 50 | 9 |

Data are presented as the number of subjects.
