# Supplementary material for: Evaluating adherence, tolerability and safety of oral calcium citrate in elderly osteopenic subjects: a real-life non-interventional, prospective, multicenter study
Source: Aging Clin Exp Res. 2024 Feb 12;36(1):38. doi: 10.1007/s40520-024-02696-9 (PMC10861607; doi:10.1007/s40520-024-02696-9)
Supplement: Supplementary file 3 — Supplementary file3 (DOCX 19 KB) [file 40520_2024_2696_MOESM3_ESM.docx]

**Supplementary Table 3**. Summary of all adverse events (episodes) related and not related to calcium citrate concentration.

| Medical Condition | | RELATED | | ALL |
| --- | --- | --- | --- | --- |
|  |  | No | Yes |  |
| Eye disorders | Scintillating scotoma | 1 (0.9%) | . | 1 (0.8%) |
|  | Visual impairment | 1 (0.9%) | . | 1 (0.8%) |
|  | All | 2 (1.8%) | . | 2 (1.5%) |
| Gastrointestinal disorders | Abdominal discomfort | 2 (1.8%) | 1 (5.0%) | 3 (2.3%) |
|  | Abdominal distension | 4 (3.6%) | . | 4 (3.1%) |
|  | Abdominal pain | 2 (1.8%) | . | 2 (1.5%) |
|  | Abdominal pain upper | 17 (15.5%) | 2 (10.0%) | 19 (14.6%) |
|  | Constipation | 15 (13.6%) | 10 (50.0%) | 25 (19.2%) |
|  | Diarrhoea | 8 (7.3%) | . | 8 (6.2%) |
|  | Dry mouth | 1 (0.9%) | . | 1 (0.8%) |
|  | Dyspepsia | 8 (7.3%) | 2 (10.0%) | 10 (7.7%) |
|  | Dysphagia | . | 1 (5.0%) | 1 (0.8%) |
|  | Flatulence | 1 (0.9%) | 1 (5.0%) | 2 (1.5%) |
|  | Gastrooesophageal reflux disease | 14 (12.7%) | . | 14 (10.8%) |
|  | Nausea | 8 (7.3%) | 2 (10.0%) | 10 (7.7%) |
|  | Regurgitation | 1 (0.9%) | . | 1 (0.8%) |
|  | Salivary hypersecretion | 1 (0.9%) | . | 1 (0.8%) |
|  | Vomiting | 5 (4.5%) | . | 5 (3.8%) |
|  | All | 87 (79.1%) | 19 (95.0%) | 106 (81.5%) |
| General disorders and administration site conditions | Adverse event | 3 (2.7%) | . | 3 (2.3%) |
|  | Condition aggravated | 1 (0.9%) | . | 1 (0.8%) |
|  | Discomfort | 1 (0.9%) | . | 1 (0.8%) |
|  | Malaise | 2 (1.8%) | . | 2 (1.5%) |
|  | All | 7 (6.4%) | . | 7 (5.4%) |
| Infections and infestations | Herpes Zoster | 1 (0.9%) | . | 1 (0.8%) |
|  | Pneumonia | 1 (0.9%) | . | 1 (0.8%) |
|  | All | 2 (1.8%) | . | 2 (1.5%) |
| Investigations | Blood pressure increased | 2 (1.8%) | . | 2 (1.5%) |
|  | Urine calcium increased | 1 (0.9%) | . | 1 (0.8%) |
|  | Weight increased | 1 (0.9%) | . | 1 (0.8%) |
|  | All | 4 (3.6%) | . | 4 (3.1%) |
| Metabolism and nutrition disorders | Decreased appetite | . | 1 (5.0%) | 1 (0.8%) |
|  | All | . | 1 (5.0%) | 1 (0.8%) |
| Musculoskeletal and connective tissue disorders | Bone pain | 2 (1.8%) | . | 2 (1.5%) |
|  | All | 2 (1.8%) | . | 2 (1.5%) |
| Nervous system disorders | Headache | 3 (2.7%) | . | 3 (2.3%) |
|  | Somnolence | 1 (0.9%) | . | 1 (0.8%) |
|  | All | 4 (3.6%) | . | 4 (3.1%) |
| Psychiatric disorders | Insomnia | 1 (0.9%) | . | 1 (0.8%) |
|  | All | 1 (0.9%) | . | 1 (0.8%) |
| Respiratory, thoracic and mediastinal disorders | Throat irritation | 1 (0.9%) | . | 1 (0.8%) |
|  | All | 1 (0.9%) | . | 1 (0.8%) |
| ALL | | 110 (100.0%) | 20 (100.0%) | 130 |

Data are presented as number (N) and percentages (%).
