# Supplementary material for: Evaluating adherence, tolerability and safety of oral calcium citrate in elderly osteopenic subjects: a real-life non-interventional, prospective, multicenter study
Source: Aging Clin Exp Res. 2024 Feb 12;36(1):38. doi: 10.1007/s40520-024-02696-9 (PMC10861607; doi:10.1007/s40520-024-02696-9)
Supplement: Supplementary file 4 — Supplementary file4 (DOCX 16 KB) [file 40520_2024_2696_MOESM4_ESM.docx]

**Supplementary Table 4.** Change from baseline in vital signs.

|  | N | Baseline | Post-Baseline | Difference | p-value |
| --- | --- | --- | --- | --- | --- |
| p-value |  |  |  |  |  |
| SBP (mmHg) | 166 | 130.7±16.9 | 127.9±14.5 | -2.8±13.9 | 0.0102 |
|  |  |  |  |  |  |
| DBP (mmHg) | 166 | 79.5±8.7 | 77.4±8.6 | -2.1±10.4 | 0.0116 |
| HR (bpm) | 149 | 76.2±9.1 | 77.1±9.4 | 0.8±9.3 | NS |
| RR (bpm) | 82 | 15.2±1.8 | 15.1±1.8 | -0.1±2.1 | NS |
| Body temperature (°C) | 84 | 36.1±0.4 | 36.1±0.4 | 0.02±0.4 | NS |
|  |  |  |  |  |  |

Data are presented as numbers of subjects (N) and mean vital signs values with relative standard deviations (SD).

Only significant p-value are reported.

SBP= Systolic Blood Pressure

DBP= Diastolic Blood Pressure

HR= Heart Rate

RR= Respiratory Rate
