# Supplementary material for: Evaluating adherence, tolerability and safety of oral calcium citrate in elderly osteopenic subjects: a real-life non-interventional, prospective, multicenter study
Source: Aging Clin Exp Res. 2024 Feb 12;36(1):38. doi: 10.1007/s40520-024-02696-9 (PMC10861607; doi:10.1007/s40520-024-02696-9)
Supplement: Supplementary file 5 — Supplementary file5 (DOCX 20 KB) [file 40520_2024_2696_MOESM5_ESM.docx]

**Supplementary Table 5.** Physical examination findings at the baseline (V1) and post-baseline (V2).

|  |  | **INTERMEDIATE/LAST VISIT (V2)** | | | | **ALL** | | **p-value** |
| --- | --- | --- | --- | --- | --- | --- | --- | --- |
|  |  | Abnormal | | Normal | |  |  |  |
| ***Baseline (V1)*** | | N | % | N | % | N | % |  |
| GENERAL CONDITIONS | *Abnormal* | 1 | 0.8% | . | . | 1 | 100.0% |  |
|  | *Normal* | . | . | 119 | 99.2% | 119 | 100.0% |  |
|  | *ALL* | 1 | 100.0% | 119 | 100.0% | 120 | 100.0% |  |
| NUTRITIONAL STATUS | *Abnormal* | 6 | 5.0% | 15 | 12.4% | 21 | 100.0% |  |
|  | *Normal* | 4 | 3.3% | 96 | 79.3% | 100 | 100.0% | **0.0116** |
|  | *ALL* | 10 | 100.0% | 111 | 100.0% | 121 | 100.0% |  |
| CIRCULATORY SYSTEM | *Abnormal* | 8 | 7.0% | 20 | 17.5% | 28 | 100.0% |  |
|  | *Normal* | 2 | 1.8% | 84 | 73.7% | 86 | 100.0% | **0.0001** |
|  | *ALL* | 10 | 100.0% | 104 | 100.0% | 114 | 100.0% |  |
| RESPIRATORY SYSTEM | *Abnormal* | . | . | 5 | 4.5% | 5 | 100.0% |  |
|  | *Normal* | . | . | 105 | 95.5% | 105 | 100.0% |  |
|  | *ALL* | . | . | 110 | 100.0% | 110 | 100.0% |  |
| ABDOMEN | *Abnormal* | . | . | 3 | 2.8% | 3 | 100.0% |  |
|  | *Normal* | 1 | 0.9% | 102 | 96.2% | 103 | 100.0% |  |
|  | *ALL* | 1 | 100.0% | 105 | 100.0% | 106 | 100.0% |  |
| LYMPH NODES | *Abnormal* | . | . | . | . | . | . |  |
|  | *Normal* |  |  | 103 | 100.0% | 103 | 100.0% |  |
|  | *ALL* |  |  | 103 | 100.0% | 103 | 100.0% |  |
| SKIN AND APPENDAGES | *Abnormal* | . | . | . | . | . | . |  |
|  | *Normal* | 1 | 0.9% | 106 | 99.1% | 107 | 100.0% |  |
|  | *ALL* | 1 | 100.0% | 106 | 100.0% | 107 | 100.0% |  |
| MUCOUS MEMBRANES | *Abnormal* | . | . | . | . | . | . |  |
|  | *Normal* | . | . | 104 | 100.0% | 104 | 100.0% |  |
|  | *ALL* | . | . | 104 | 100.0% | 104 | 100.0% |  |
| MUSCLES / SKELETON | *Abnormal* | 17 | 15.9% | 10 | 9.3% | 27 | 100.0% | **0.0067** |
|  | *Normal* | 1 | 0.9% | 79 | 73.8% | 80 | 100.0% |  |
|  | *ALL* | 18 | 100.0% | 89 | 100.0% | 107 | 100.0% |  |
| NEUROLOGICAL SYSTEM | *Abnormal* | 1 | 1.2% | . | . | 1 | 100.0% |  |
|  | *Normal* | . | . | 85 | 98.8% | 85 | 100.0% |  |
|  | *ALL* | 1 | 100.0% | 85 | 100.0% | 86 | 100.0% |  |

Data are presented as number of subjects (N) and percentage (%).

Only significant changes between V1 and V2, calculated using McNemar test, are shown.
